# Editorial: Highlights of iMMM2023 - International Molecular Mycorrhiza Meeting

**DOI:** 10.3389/fpls.2025.1559814

**Published:** 2025-02-03

**Authors:** Marcel Bucher, Andrea Genre, Hiromu Kameoka, Luisa Lanfranco, Uta Paszkowski, Li Xue

**Affiliations:** ^1^ Institute for Plant Sciences, Cologne Biocenter, Cluster of Excellence on Plant Sciences, University of Cologne, Cologne, Germany; ^2^ Department of Life Science and Systems Biology, University of Turin, Turin, Italy; ^3^ Chinese Academy of Sciences - John Innes Centre (CAS-JIC) Centre of Excellence for Plant and Microbial Science (CEPAMS), Center for Excellence in Molecular Plant Sciences (CEMPS), Chinese Academy of Sciences, Shanghai, China; ^4^ Department of Plant Sciences, University of Cambridge, Cambridge, United Kingdom; ^5^ Shandong Agricultural University, Taian, China

**Keywords:** symbiosis, arbuscular mycorrhiza, ectomycorrhiza, ericoid mycorrhiza, orchid mycorrhiza

Mycorrhizas are common mutualistic symbioses formed between soil fungi and plant roots. The symbiotic status improves plant mineral nutrition at the cost of a fraction of the photosynthetically fixed carbon. As a result, plant growth is positively impacted as well as host resistance to biotic and abiotic stresses. Furthermore, mycorrhizas provide a number of ecosystem services in both agricultural and natural settings. Indeed, mycorrhizal fungi shape microbial and plant communities, enhance carbon storage, and alter soil particle aggregation (Chen et al., 2018; Tedersoo et al., 2020).

Arbuscular, ecto–, orchid, and ericoid mycorrhizas are the four main mycorrhizal types, each with distinct morphological and functional traits resulting from over 400 million years of co-evolution between plants and symbiotic fungi (Genre et al., 2020). Over 320,000 existing vascular and non-vascular plant species can develop mycorrhizas, with the largest and most varied group of species belonging to angiosperms. Trees, bushes, herbs, and most staple crops (including rice, maize and tomato) are among them. Within this astounding diversity, arbuscular mycorrhizas are of particular interest due to their potential to support sustainable crop production in the context of global climate change. Ectomycorrhizas have massive potential in forest management, while ericoid and orchid mycorrhizas have successfully been applied in bioremediation and ecosystem conservation studies.

Launched in conjunction with the 6^th^ International Molecular Mycorrhiza Meeting (iMMM 2023), which took place in Cambridge, UK, from September 25–27, 2023, this Research Topic has yielded seven selected contributions that cover most of the topics discussed at the meeting with original research, methods and review papers about mycorrhizal associations. In line with the meeting’s major focus, most studies dealt with molecular aspects of arbuscular mycorrhizal interactions.

*Parasponia andersonii* (*Cannabaceae*) serves as a unique model system for studying plant symbioses, being the only non-legume capable of forming nitrogen-fixing nodules with rhizobia (Dupin et al., 2020). Sharing core symbiotic genes with legumes yet diverging over 100 million years ago, *Parasponia* offers a rare opportunity to compare evolutionary pathways of symbiotic interactions. Its genetic tractability and ability to engage in both arbuscular mycorrhizal (AM) and nodulation symbioses make it a valuable research tool. The study by Alhusayni et al. explores the GRAS-type transcriptional regulator NSP2 in *Parasponia*, highlighting its dual role in symbiotic processes. Ectopic overexpression of NSP2 enhances AM colonization and strigolactone biosynthesis, even under high phosphate conditions, but at a cost. Pleiotropic effects include reduced lateral root formation, increased shoot branching, and irregular cell divisions upon rhizobia inoculation, negatively impacting nodulation. The findings underscore the complexity of using NSP2 in agricultural biotechnology and emphasize the need for balanced expression strategies to optimize benefits while minimizing developmental trade-offs.

The review by He et al. focuses on host plant recognition of mycorrhizal factors, specifically lipo-chitooligosaccharides (LCOs) and chitooligosaccharides (COs), through LysM-type receptors. The authors introduce the concept of a mycorrhizal biceptor complex, playing a role in plant detection of both LCOs and COs. The review explores the molecular mechanisms of receptor function and suggests future research directions, particularly for improving AM fungi utilization in cereal crops like rice, to address agricultural challenges.

The exploration of AM fungal genomes highlighted hundreds of genes encoding putative effectors that are thought to be delivered towards the plant cells to modify plant immune responses favoring fungal accommodation and the establishment of a functional symbiosis. Understanding the role of these effectors is challenging because of the obligate biotrophy and recalcitrance to genetic transformation of AM fungi. To overcome these issues, by using a combination of different biological systems and different biochemical and genetic approaches, Aparicio Chacón et al. performed the characterization of four putative effectors from the model AM fungus *Rhizophagus irregularis*. These proteins, localized in the plant cell nucleus, have an impact on plant growth and mycorrhizal colonization when expressed ectopically in the host plant. The work is of particular relevance because it also offers a critical analysis of the experimental tools used.

A methodological advancement was proposed by Jarratt-Barnham et al., who developed two tools to streamline the analysis of AM colonization in plants, a critical task for many researchers in the field. *AMScorer* simplifies the process of recording and processing microscopy-based data, significantly reducing data collection time. Building on this, *AMReader* allows for easy visualization and statistical analysis of the data. Both tools require minimal expertise in Excel and R and are freely available for download with a detailed user manual.

A further study was devoted to ecological aspects of AM symbiosis, with the work by Guillen-Otero et al., who explored the relationship between mycorrhizal fungi and the fern *Struthiopteris* sp*icant* across Europe and North America, focusing on how environmental factors influence mycorrhizal community composition. Using a metabarcoding approach, the researchers found significant variation in AM fungal abundance among fern populations. In this frame, only a small portion of fungal diversity was shared across individuals, suggesting each fern hosts a unique fungal community. The limited effect of environmental factors supports the idea that ferns interact with mycorrhizal fungi in a more opportunistic manner, compared to other plants.

Lastly, two papers provided original insights into other types of mycorrhizal interactions. The brief report by Ho-Plágaro et al. investigates the role of the *HcZnT2* gene in the ectomycorrhizal fungus *Hebeloma cylindrosporum*, which is involved in regulating zinc (Zn) transport to plants. *HcZnT2* is upregulated since early fungal interaction with the host plant and repressed under elevated zinc conditions. Functional experiments suggest a key role in zinc homeostasis during the early stages of ectomycorrhizal symbiosis.

The research by Pujasatria et al. focuses on the induction of plant resistance to pathogens by orchid mycorrhiza (OM). AM is formed between most terrestrial plants and fungi in the subphylum Glomeromycotina, whereas OM is formed between Orchidaceae and certain fungi from the phylum Basidiomycota or, occasionally, Ascomycota. It has been shown that AM enhances plant resistance to pathogens, a phenomenon known as induced systemic resistance (ISR). While the mechanism of ISR induced by AM has been well studied, only few studies have investigated ISR induced by OM. This study confirmed that OM induces ISR and, through transcriptome analysis, suggested that the jasmonic acid and ethylene pathways are involved in OM-induced ISR, similar to what has been observed in AM.

As we are writing this Editorial, the IMMM 2023 Research Topic has gained more than 13,000 views and 4,000 downloads. This success mirrors the vigour of the iMMM community and the increasing interest in mycorrhizal interactions both for their applicative potential and their uniqueness as biological models.
